# Current overview and treatment of mantle cell lymphoma

**DOI:** 10.12688/f1000research.14122.1

**Published:** 2018-07-25

**Authors:** Michael Schieber, Leo I. Gordon, Reem Karmali

**Affiliations:** 1 Robert H. Lurie Comprehensive Cancer Center of Northwestern University, Chicago, Illinois, USA

**Keywords:** Mantle Cell Lymphoma, Management, Treatment, Diagnosis, Clinical trials

## Abstract

Mantle cell lymphoma (MCL) is a B-cell non-Hodgkin lymphoma with historically poor long-term survival compared with other B-cell malignancies. Treatment strategies for this disease are variable and dependent on symptoms and patient fitness. Despite recent advances, MCL remains incurable and patients with high-risk disease have particularly poor outcomes. This review focuses on recent developments that enhance our understanding of the biology of MCL and new treatment approaches that have led to substantial improvements in clinical outcomes. We will outline induction immuno-chemotherapy and maintenance strategies in transplant-eligible patients. In addition, effective strategies for patients unfit for intensive induction will be discussed, with a particular focus on novel molecular therapies with activity in MCL. Lastly, a number of ongoing clinical trials will be presented; the data from these trials are anticipated to redefine standards of care in the near future.

## Introduction

Mantle cell lymphoma (MCL) is an aggressive mature B-cell non-Hodgkin lymphoma (B-NHL) with historically poor long-term survival. Recent progress in our understanding of the biology of MCL has led to substantial improvements in patient outcomes and the development of a number of novel targeted therapies. In this review, the pathogenesis of MCL will be reviewed with a focus on newly defined MCL subtypes that predict response to therapy. We will discuss the major breakthroughs in MCL care in the past 5 years, including recent phase III clinical data that reinforce the use of high-dose cytarabine induction immunochemotherapy in fit patients. There are now data from randomized studies supporting the use of rituximab maintenance in MCL post-autologous stem cell transplant (ASCT). A number of new strategies for the management of patients unfit for ASCT will be reviewed. For those patients who unfortunately experience a relapse of their disease, a number of novel therapies have demonstrated substantial benefit, most notably the Bruton’s tyrosine kinase (BTK) inhibitors ibrutinib and acalabrutinib. Lastly, mechanisms of therapy resistance as well as future directions of treatment will be discussed.

## Biology of mantle cell lymphoma

While defined as a mature B-NHL, MCL has a clinical presentation and natural course that often mimic its more aggressive B-NHL counterparts. Patients commonly have lymphadenopathy, splenomegaly, bone marrow involvement, and gastrointestinal infiltration at the time of diagnosis. MCL is classically defined by the t(11;14)(q13;q32) translocation which juxtaposes the *CCDN1* gene encoding cyclin D1 to the immunoglobulin heavy chain (IgH), resulting in the overexpression of cyclin D1. Alternatively, less-frequent alterations in *CCND2* and *CCND3*, encoding cyclin D2 and D3, respectively, have been identified in MCL lacking the t(11;14)(q13;q32) translocation ^1^. However, over the past decade, further investigation into the pathogenesis of MCL has defined other molecular abnormalities that define cyclin D1-negative MCL.

SOX11, a SOX family transcription factor with a role in cell fate and differentiation, has been identified as a reliable diagnostic and prognostic marker of MCL in both cyclin D1-positive and -negative disease ^2^. Microarray analyses and protein expression quantification by immunohistochemistry (IHC) have shown that SOX11 overexpression is an independent molecular feature of MCL regardless of cyclin D1 status. Furthermore, a complex transcription signature accompanies the overexpression of SOX11. This includes the regulation of the transcription factor PAX5, which is critical for late B-cell differentiation ^3^. Increased PAX5 levels repress the key transcriptional regulators of plasma cell differentiation BLIMP1, XB1, and IRF4. Supporting this, increased SOX11 expression correlates with an unmutated or minimally mutated *IGHV* genotype, a marker of germinal center maturation and differentiation into memory B-cells ^4^. Loss of SOX11 expression, and subsequent downregulation of PAX5, correlates with a hypermutated *IGHV* genotype, reflective of early steps towards plasma cell differentiation. Clinically, low SOX11 expression correlates with a more indolent and non-nodal leukemic phase presentation, whereas increased SOX11 expression portends a more aggressive phenotype with nodal and extra-nodal sites of involvement. Taken together, the implementation of *CCND2, CCND3,* SOX11, and *IGHV* analysis has expanded criteria for the molecular diagnosis of MCL.

## Prognostic variables and observation in mantle cell lymphoma

MCL is thought to be an aggressive disease in most patients, associated with early relapse and poor long-term survival. Standard of care strategies continue to emphasize aggressive treatment approaches, which have demonstrated the longest durable remissions. However, observation can be considered in patients with favorable disease features ^5, 6^. Easily obtained clinical variables provide excellent risk stratification of patients. The MCL International Prognostic Index (MIPI) incorporates patient age, performance status, lactate dehydrogenase (LDH), and white blood cell count into a formula to stratify patients as low, intermediate, or high risk; 5-year overall survival for MIPI-low patients was 83% compared with 34% in MIPI-high patients, with a high MIPI score also predicting inferior response to therapy ^7^. A similar tool, the Biologic MIPI, incorporates the Ki-67 proliferation rate, usually readily obtained from pathology specimens, to better identify high-risk disease ^8^.

Among the various clinical and pathologic factors analyzed in a population-based study reported by the British Columbia Cancer Agency (n=440), non-nodal presentation, defined as patients presenting with lymphocytosis and/or splenomegaly, was the single factor most strongly associated with prolonged time to treatment and excellent overall survival. In this study, median time to treatment was 35 months without a negative impact on overall survival in patients deemed suitable for initial observation. Other characteristics of the observed patients included good performance status, absence of B-symptoms, non-blastoid morphology, and Ki67 percentage of less than 30%. There were no significant differences in the observed versus treatment groups, including in age at diagnosis, sex, leukocyte count, platelet count, stage, TP53 and SOX11 expression by IHC, and MIPI risk score ^5^.

While there are no prospective data guiding observation in patients based on SOX11 or TP53 expression, these are additional variables that can be considered clinically to predict disease course. In the British Columbia study above, P53 positivity was defined as strong nuclear staining and SOX11 was positive if any cells stained positive. A second study by the European MCL Network sorted patients into groups based on percentage of P53-positive cells (1–10%, 10–50%, and over 50%) and SOX11 cells (0%, 1–10%, and over 10%) by IHC ^9^. In this study, the cohort with over 50% TP53 expression had poorer prognosis, while SOX11 expression was not a reliable prognostic marker, possibly owing to an under-representation of patients with non-nodal disease. Another explanation is a difference in methods to determine SOX11 positivity, as Navarro *et al*. used a combination of reverse transcriptase polymerase chain reaction (RT-PCR) to measure SOX11 RNA, IHC to measure SOX11 protein, and gene expression profiling and observed an improved clinical outcome with low SOX11 expression.

It is important to note that no patients in the above cohorts were observed with the pathologic blastoid variant of MCL, which continues to be a clinical challenge ^10^. This group, which comprises less than 20% of total MCL diagnoses, features medium-sized lymphocytes with indistinct cytoplasm and dispersed chromatin compared with classical-type MCL with smaller lymphocytes and condensed chromatin. In a subgroup analysis of all modern trials, the patients with blastoid phenotype have inferior survival and remission rates. A further understanding of the disease process in these patients is greatly needed for the development of novel therapies. Thus, when deciding on initial observations, we consider all of the aforementioned disease features and balance this with the patient’s preferences and other comorbidities. It should be remembered that many patients will not behave as their risk features predict, so close monitoring for progression of disease is necessary in all observed patients.

## Treatment strategies in fit patients

### Induction chemotherapy

Once the decision is made to proceed with treatment, the oncologist must determine whether or not the patient is fit for intensive therapy. Treatment broadly consists of two components: cytarabine-containing induction chemotherapy followed by ASCT. Phase II trials in the past decade have consistently demonstrated the impressive activity of cytarabine in MCL, namely in the Hyper-CVAD and VcR-CVAD regimens ^11– 13^. Since that time, the MCL Younger trial conducted by the European MCL Network provided phase III data to support cytarabine as the key agent in MCL induction ^14^. This trial included patients aged 65 years or younger with stage II–IV MCL randomized to six cycles of R-CHOP (non-cytarabine group) versus alternating R-CHOP and R-DHAP for six cycles (cytarabine group). Patients then proceeded to ASCT with myeloablative conditioning. Time to treatment failure in the cytarabine group was 9.1 years compared with 3.9 years in the non-cytarabine cohort, a benefit attributed to deeper molecular responses in patients receiving cytarabine. This benefit came at the expense of higher hematologic and gastrointestinal toxicities. The impressive results of the MCL Younger trial have redefined the standard treatment paradigm for fit patients with MCL but also raise a number of interesting questions going forward. Ongoing long-term follow-up of the trial will demonstrate whether cytarabine immunochemotherapy results in treatment cures in certain patients, although long-term follow-up of the Nordic trial suggests that this may still not be the case ^15, 16^. Additionally, it is unclear how much benefit cytarabine immunochemotherapy provides for blastoid-variant MCL, a cohort that made up less than 10% of the population in the MCL Younger trial. Similarly, among the 5% of patients with a high combined MIPI and Ki-67 expression score treated in the European MCL Network Younger trial, median overall survival was approximately 2 years ^8^. Each of these patient subsets still perform poorly, even with modern cytarabine-containing regimens and/or ASCT, suggesting that novel treatment strategies are needed for these groups.

The use of ASCT consolidation in first remission is supported by data published by the European and Nordic groups who noted significantly prolonged progression-free survival (PFS) with ASCT ^15^, with the European group randomizing patients to interferon versus ASCT ^17^. However, these data were attained before the widespread use of cytarabine induction regimens, maintenance rituximab in first remission, and the discovery of BTK inhibitors. Thus, randomized data confirming the efficacy of ASCT are greatly needed given the number of novel strategies recently developed in MCL. The European MCL Network phase III TRIANGLE study is currently randomizing patients to an induction regimen containing the BTK inhibitor ibrutinib while also assessing whether an ibrutinib-containing induction regimen with maintenance can replace ASCT. This will be the first phase III trial to incorporate a targeted molecular therapy into the MCL induction regimen and also the first randomized study to test the efficacy of ASCT in the cytarabine and rituximab era.

### Post-transplant maintenance and surveillance

While most patients have no evidence of disease post-ASCT, the high relapse rate of MCL supports the concept of undetectable minimal residual disease (MRD) that precedes the development of a clinical relapse. This has been most rigorously studied by the Nordic MCL Group, who have measured MRD both pre- and post-ASCT by designing individualized RT-PCR primers to detect clonal *IgH* rearrangements on bone marrow and peripheral blood samples ^18^. Seroconversion to positive RT-PCR or a fivefold increase in RT-PCR MRD levels was defined as a molecular relapse (but not counted as a clinical relapse). Followed prospectively, PFS in MRD-negative patients was 142 months compared with 35 months in MRD-positive patients. This improvement in PFS also correlated with an impressive overall survival advantage. While certainly a proof of concept, there is currently no wide-scale approach for measuring MRD in large numbers of patients, and these methods will require validation across more institutions. The absence of such a universal approach has prompted the investigation of maintenance rituximab to produce more durable long-term remissions post-ASCT ^19^. The Nordic group first showed that pre-emptive treatment with four doses of rituximab at first sign of molecular relapse led to a re-induction of molecular remission in over 90% of patients ^20^. Prospective phase III data to support maintenance rituximab have become available with the recent published results of the multicenter LyMa trial conducted in France ^21^. Patients were randomized in a 1:1 ratio, irrespective of MRD status, to observation versus 3 years of maintenance rituximab after four cycles of R-DHAP induction followed by ASCT. PFS at 4 years was 79% in the rituximab group versus 61% with observation alone. Importantly, rituximab maintenance provided a statistically significant overall survival advantage and thus should be considered a standard of care post-ASCT. What remains unanswered, however, is whether consolidative ASCT provides any additional benefit in the rituximab maintenance era. A major advance towards this question is currently underway in a phase III ECOG trial that is randomizing patients without MRD to autologous transplant with maintenance rituximab to rituximab alone ( Table 1). Additionally, investigation into the feasibility and efficacy of ibrutinib as an alternative for (or adjuct to) rituximab and as a replacement for ASCT will be addressed in the phase III TRIANGLE study and a phase II trial currently enrolling at our institution ( Table 1).

**Table 1.** Current clinical trials in mantle cell lymphoma (MCL). Notable current clinical trials in MCL organized by clinical indication. Certain trials appear twice given their design to answer multiple clinical questions. ASCT, autologous stem cell transplant; BCL-2, B-cell lymphoma 2; BR, bendamustine–rituximab; BTK, Bruton’s tyrosine kinase; CAR-T; chimeric antigen receptor T-cell; HDAC, histone deacetylase; R-Hyper-CVAD, rituximab plus fractionated cyclophosphamide, vincristine, doxorubicin, and dexamethasone plus methotrexate and cytarabine; MRD, minimal residual disease; PI3K, phosphoinositide 3-kinase; R ^2^, lenalidomide plus rituximab; R-CHOP, rituximab, cyclophosphamide, doxorubicin, vincristine, and prednisolone; R-DHAP, rituximab, dexamethasone, cytarabine, and cisplatin; R-HAD, rituximab, high-dose cytarabine, and dexamethasone.

Category of therapy	Study design or regimen	Target or drug class	Clinical trials identifier	Status	Phase
**Fit induction**	R-CHOP/R-DHAP + ASCT vs. R-CHOP/R-DHAP + ibrutinib vs. R-CHOP/R-DHAP + ibrutinib + ASCT	BTK	NCT02858258 (TRIANGLE)	Recruiting	Phase III
	R-HyperCVAD + Ibrutinib	BTK	NCT02427620	Recruiting	Phase II
**Unfit induction**	BR + ibrutinib	BTK	NCT01776840 (SHINE)	Active	Phase III
	R-CHOP/R-HAD vs. R-CHOP	Cytarabine	NCT01865110	Recruiting	Phase III
	BR + acalabrutinib	BTK	NCT02972840	Recruiting	Phase III
	Bendamustine + obinutuzumab	Anti-CD20	NCT03311126	Recruiting	Phase II
**Maintenance**	Rituximab vs. ASCT + rituximab in MRD-negative patients	Anti-CD20	NCT03267433	Recruiting	Phase III
	ASCT +/- ibrutinib maintenance vs ibrutinib maintenance (no ASCT); rituximab may be added to each arm	BTK	NCT02858258 (TRIANGLE)	Recruiting	Phase III
	Ibrutinib without ASCT	BTK	NCT02242097	Recruiting	Phase II
	R ^2^ vs. rituximab maintenance	Anti-CD20 + Imid	NCT01865110	Recruiting	Phase III
**Relapsed MCL**	Obinutuzumab + GDC-0199 + Ibrutinib	Anti-CD20, BCL-2, BTK	NCT02558816	Recruiting	Phase II
	Obinutuzumab + Ibrutinib	Anti-CD20 + BTK	NCT02736617	Recruiting	Phase II
	KTE-C19	CAR-T	NCT02601313 (ZUMA-2)	Recruiting	Phase II
	JCAR017	CAR-T	NCT02631044 (TRANSCEND)	Recruiting	Phase I
	Ixazomib + Ibrutinib	Proteasome + BTK	NCT03323151	Recruiting	Phase II
	Bortezomib + Ibrutinib	Proteasome + BTK	NCT02356458	Recruiting	Phase II
	INCB050465	PI3K	NCT03235544	Recruiting	Phase II
	Entospletinib	Syc	NCT01799889	Active	Phase II
	Vorinostat	HDAC	NCT00875056	Recruiting	Phase II
	Enzalutamide	Androgen	NCT02489123	Recruiting	Phase II

It is important to analyze the results of the LyMa trial in the context of the MCL Younger trial. The LyMa trial produced impressive results with an anthracycline and alkylating-free induction regimen. Patients who failed to respond to four cycles of R-DHAP proceeded to salvage R-CHOP prior to transplant. This stepwise progression spared over 90% of the study participants from additional cytotoxic chemotherapy exposure and their associated long-term toxicities.

## Therapy strategies in unfit patients

The treatment paradigm in MCL changes when patient preferences or comorbidities preclude intensive therapies including ASCT. In this scenario, clinical judgment should drive the safest and most-effective induction chemotherapy regimen. A phase III randomized trial established superiority of R-CHOP over fludarabine plus cyclophosphamide in unfit patients with a median age of 70 and confirmed the benefit of lifetime rituximab maintenance in responders ^22^. Since this study, a number of regimens have shown marked improvement in clinical outcomes over R-CHOP ( Table 2). MCL patients comprised approximately 15–20% of the total treated population in two recent phase III studies demonstrating non-inferiority of bendamustine–rituximab (BR) to R-CHOP ^23, 24^. BR demonstrated an improved safety profile and PFS that carried through into the MCL subgroup analysis. It should be noted that while these trials were designed for elderly patients with non-aggressive NHL, the median age difference compared with the MCL Younger study was a modest 5–10 years. Therefore, as is probably true in oncology in general, the decision of intensive treatment should be based more on performance status and goals of therapy than absolute age alone.

**Table 2.** Regimens for mantle cell lymphoma (MCL). This table presents treatment regimens for MCL organized by clinical indication. BR, bendamustine–rituximab; CRR, complete response rate; CrU, unconfirmed complete response rate; LBR, lenalidomide, bendamustine, and rituximab; ORR, overall response rate; NR, not reported; R ^2^, lenalidomide plus rituximab; R-BAC(500), rituximab, bendamustine, and cytarabine with low-dose cytarabine; R-CHOP, rituximab, cyclophosphamide, doxorubicin, vincristine, and prednisolone; R-DHAP, rituximab, dexamethasone, cytarabine, and cisplatin; R-Hyper-CVAD, rituximab plus fractionated cyclophosphamide, vincristine, doxorubicin, and dexamethasone; RiBVD, rituximab, bendamustine, bortezomib, and dexamethasone; VcR-CVAD, bortezomib, rituximab, hyper-fractionated cyclophosphamide, vincristine, doxorubicin, and dexamethasone; VR-CAP, bortezomib, rituximab, cyclophosphamide, doxorubicin, and prednisone.

Treatment category	Regimen	Number of patients	ORR	CRR (CrU)	Reference
**Fit patients**	R-Hyper-CVAD	97	97%	77% (87%)	12
	VcR-CVAD	75	95%	68%	11
	R-DHAP x 4	299	89%	41% (77%)	20
	R-DHAP/R-CHOP x 6	248	94%	55% (91%)	13
**Unfit patients**	BR	50	94%	50%	22
	RiBVD	74	86%	NR (74%)	24
	LBR	50	88%	32% (64%)	25
	R ^2^	38	92%	64%	26
	VR-CAP	229	92%	53%	27
	R-BAC(500)	57	NR	91%	28
**Relapsed disease**	Bortezomib	155	33%	6% (8%)	29
	Temsirolimus	54	22%	2%	30
	Lenolidamide	170	78%	19% (11%)	31
	Ibrutinib	139	72%	19%	8
	Acalabrutinib	124	81%	40%	32
	Ibrutinib + Venetoclax	24	71%	62%	33

A number of studies have attempted to improve the efficacy of the BR backbone in elderly patients by including agents which have shown efficacy in relapsed disease. These include phase II data demonstrating the efficacy of adding bortezomib, dexamethasone, and lenalidomide (RiBVD) ^25^, lenalidomide (LBR) ^28^, or cytarabine (R-BAC500) ^26^. The results of the phase III SHINE study combining BR with ibrutinib should be available in the upcoming year. It is also possible to spare certain patients alkylating chemotherapy, as an 85% PFS was achieved with a 12-cycle regimen of lenalidomide plus rituximab (R ^2^) ^27^. Improved PFS was observed in the phase III LYM-3002 study by substituting bortezomib for vincristine in the VR-CAP regimen in a patient population with a median age of 65 ^29^. Given these results, our preference has been to utilize these newer regimens with rituximab maintenance over traditional R-CHOP alone in unfit patients, with options summarized in Table 2, tailored to patients based on tolerability, comorbid conditions, and performance status.

As mentioned above, based on the success of cytarabine-containing induction regimens in younger fit patients, the Italian MCL group studied its addition to a BR backbone in elderly patients (R-BAC500) ^26^. Rather than three doses at 2 g/m ^2^ in the MCL younger trial (D-HAP regimen), the investigators lowered the cytarabine dose to three doses at 500 mg/m ^2^ per cycle. This produced an acceptable hematological toxicity profile that allowed 95% of patients to complete four cycles of therapy. PFS was approximately 75% at 3 years in a patient population with a median age of 71, an impressive result given rituximab maintenance was not included in the protocol. These results warrant confirmation in a randomized setting and may prove to be the next major advance to sustainable remissions in the elderly population.

## Relapsed mantle cell lymphoma

Relapsed MCL has historically been characterized by poor response rates and an overall survival of less than 3 years. However, the past decade has seen multiple agents approved with single agent efficacy in relapsed disease. First, the proteasome inhibitor bortezomib received FDA approval in MCL based upon phase II clinical data demonstrating a median PFS of approximately 6 months ^30^. A multicenter open-label phase III study demonstrated superiority of temsirolimus to investigator’s choice therapy with a median PFS of 4.8 months, leading to its approval in Europe ^34^. Third, the phase II EMERGE trial confirmed the activity of lenalidomide in patients with progression of disease on bortezomib, with an overall response rate (ORR) of 28% in a heavily pre-treated population and durable efficacy with long-term follow-up, leading to lenalidomide’s approval in the US ^31, 35^. Subsequently, the SPRINT trial conducted by the European MCL Network randomized 254 patients to receive lenalidomide versus investigator’s choice treatment and found a median PFS of 8.7 months in the lenalidomide group compared with 5.2 months in the control population ^36^. In an international phase II trial in relapsed MCL patients treated with single-agent ibrutinib and included patients previously exposed to bortezomib ^32^, a 17.5-month PFS survival was observed. Lastly, pre-existing atrial fibrillation and need for anticoagulation has limited the use of ibrutinib in certain patients and led to the development and approval of the selective BTK inhibitor acalabrutinib. Phase II data have shown a favorable safety profile and durable remissions with this agent, but its non-inferiority or superiority to ibrutinib has not been tested ^37^. Nonetheless, this agent has also recently received FDA approval for relapsed/refractory MCL.

The impressive PFS of single-agent ibrutinib prompted a European phase III multicenter study comparing single-agent ibrutinib with temsirolimus ^33^. Median PFS in patients with ibrutinib was 14.2 months compared with 6.2 months in the temsirolimus cohort, confirming prior phase II data. Importantly, ibrutinib was associated with fewer treatment-related adverse effects. While the difference in overall survival was not significant between the two groups, 23% of patients assigned to temsirolimus subsequently received ibrutinib at relapse, perhaps confounding the overall survival data. This study is the highest-quality evidence supporting ibrutinib as standard of care in patients with relapsed MCL.

Multiple efforts are now underway to combine the small molecule inhibitors with single agent activity in relapsed and refractory MCL ( Table 1). Most recently, promising phase II data combining ibrutinib and the BCL-2 inhibitor venetoclax in 24 patients achieved a 42% complete response rate (CRR) compared with a 9% CRR in historical controls ^38, 39^. The efficacy and safety of this combination will have to be confirmed in a larger cohort, but this is an early indication that synergism between single agents may be a fruitful strategy in this disease.

For patients who unfortunately fail to respond to the traditional and novel therapies, allogeneic stem cell transplant (allo-SCT) remains a salvage option for disease control ^40, 41^. It is difficult to know the exact benefit of allo-SCT given the lack of randomized data in a population with few alternative options. However, the German group produced 5-year PFS rates of 67% in this high-risk group with plateauing survival curves that suggest some of these patients may be cured. This benefit, not surprisingly, comes at the cost of a treatment-related mortality rate of approximately 25%. As in all cases of allo-SCT, a referral to a high-volume transplant center is key for the best outcome.

## New insights into mantle cell lymphoma pathogenesis and future therapies

Despite the promising single agent efficacy of ibrutinib, more options are badly needed for patients with relapsed disease given the 1-year overall survival rate of approximately 70% in the ibrutinib era ^33^. Recent studies into mechanisms for ibrutinib resistance have identified the upregulation of NF-κB signaling as an adaptive mechanism to bypass the antigen receptor B-cell signaling inhibited by ibrutinib ^42, 43^. The bromodomain family of proteins are transcriptional enhancers that are required for NF-κB signaling and thus are an attractive target in MCL. Bromodomain antagonists have been shown to work synergistically with ibrutinib *in vitro* to induce apoptosis in MCL cell lines ^44, 45^. Further studies are awaited to assess whether these agents can be safely used *in vivo* alone or with ibrutinib.

The recent approval of chimeric antigen receptor T-cell (CAR-T) therapy in large cell lymphoma has opened up a novel line of therapy for patients. Indeed, a phase II study of CAR-T therapy in relapsed MCL is currently underway with an identical CD19 antigen receptor ( Table 1). As practitioners gain more experience with the administration of these products, there will be great interest in testing their efficacy both in a relapsed setting and as part of upfront therapy versus consolidative ASCT.

The male to female predominance of approximately 4:1 in MCL led a group of investigators to examine androgen receptor (AR) expression in MCL cell lines ^46^. Interestingly, compared with non-MCL cell lines, MCL cells demonstrate increased AR gene expression and elevated PSA levels consistent with active AR signaling. AR blockade with enzalutamide, an anti-androgen currently FDA approved in prostate cancer, decreased MCL cell proliferation, prompting the opening of a phase II trial to clinically investigate this effect ( Table 1).

Further investigation into the molecularly defined subtypes of MCL has raised the possibility that the treatment might be tailored based on these results. For example, can a non-nodal *SOX11*-negative patient be managed without intensive chemotherapy induction or consolidative ASCT? Additional genetic abnormalities are still being discovered and will likely impact the risk stratification of patients and treatment strategies. For example, ataxia-telangiectasia mutated (ATM) is altered in approximately 50% of MCL cases. This protein functions as a sensor for DNA damage and, while overall it does not impact long-term survival, it may sensitize MCL cells to DNA-damaging therapy or ionizing radiation ^47^. A recent shotgun sequencing approach identified 12% of patients with NOTCH1 mutations that correlated with sensitivity of MCL cells to NOTCH inhibition *in vitro* ^48^. Given the poorer prognosis of these patients, exploring drugs that target this particular mutation is appealing. TP53 mutations portend a dismal prognosis in MCL, and analysis of the TP53 cohort from the Nordic MCL2 and MCL3 trials suggests that these patients do not benefit from cytarabine-containing induction chemotherapy or ASCT ^49^. Enrichment of these patients in clinical trials using recently approved small molecule inhibitors is needed.

Given the number of potential therapies on the horizon in MCL, continued patient enrollment in clinical trials is of the utmost importance. Patients who are fit and unfit for induction as well as patients with relapsed disease should be encouraged to participate, as previous MCL trials have been criticized for their bias for healthier patients ^50^. This can not only confound non-randomized phase II efficacy data but also underestimate the side effect and toxicity data of newer regimens as they are applied to a more representative population.

## Conclusions

The exciting developments in MCL over the past decade have begun to make substantial improvements in patient quality of life and overall survival. Ongoing long-term follow-up of the most recent clinical data will hopefully provide further evidence of durable remissions and acceptable long-term side effect profiles. We await new data incorporating our newest therapies, such as ibrutinib, with induction cytarabine chemotherapy regimens to possibly eliminate the need for upfront ASCT. The optimal duration of rituximab maintenance post-induction and whether ibrutinib maintenance is a safe and efficacious alternative to rituximab also remain unanswered. Despite these recent advances, our new therapies still fail in many patients. The continued development of targeted molecular signaling inhibitors based on the underlying biology of MCL is a therapeutic approach that will continue to yield fruitful results in this disease.
